# Developing an Amide-Spacered Triterpenoid Rhodamine Hybrid of Nano-Molar Cytotoxicity Combined with Excellent Tumor Cell/Non-Tumor Cell Selectivity

**DOI:** 10.3390/molecules28176404

**Published:** 2023-09-01

**Authors:** Niels V. Heise, Toni C. Denner, Selina Becker, Sophie Hoenke, René Csuk

**Affiliations:** NF II, Organic Chemistry, Martin-Luther-University Halle-Wittenberg, Kurt-Mothes-Str. 2, 06120 Halle (Saale), Germany; niels.heise@chemie.uni-halle.de (N.V.H.); toni-christopher.denner@chemie.uni-halle.de (T.C.D.); selinabecker11@googlemail.com (S.B.); sophie.hoenke@chemie.uni-halle.de (S.H.)

**Keywords:** asiatic acid, rhodamine conjugates, cytotoxicity

## Abstract

Asiatic acid, a pentacyclic triterpene, was converted into a series of piperazinyl, homopiperazinyl, and 1,5-diazocinyl spacered rhodamine conjugates, differing in the type of spacer and the substitution pattern on the rhodamine moiety of the hybrids. The compounds were tested for cytotoxic activity in SRB assays and compound **12**, holding an EC_50_ of 0.8 nM, was the most cytotoxic compound of this series, but compound **18** (containing a ring expanded 1,5-diazocinyl moiety and *n*-propyl substituents on the rhodamine) was the most selective compound exhibiting a selectivity factor of almost 190 while retaining high cytotoxicity (EC_50_ = 1.9 nM, for A2780 ovarian carcinoma).

## 1. Introduction

Currently, a range of methodologies are being investigated for the targeted transportation of biologically active molecules and medicinal agents to mitochondria. One such approach involves the coupling of biologically active substances with cations from lipophilic compounds of modest molecular mass, which are predisposed to accumulation within mitochondria. This facilitation of entry into mitochondria is attributed to the heightened transmembrane potential relative to the cellular membrane potential. The facile traversal of these cationic entities through membranes has undergone comprehensive scrutiny and is explicable by the expansive hydrophobic surface and substantial ionic radius exhibited by these cations. Among the delocalized lipophilic cations that traverse the hydrophobic barriers of both plasma and mitochondrial membranes are Rhodamine-123, rhodacyanine MKT-077, dequalinium, triphenylphosphonium, guanidinium cations, and the recently uncovered cationic entity F16. Previous studies have shown acetylated conjugates of triterpene carboxylic acids with secondary cyclic amines and rhodamine B or rhodamine 101 in the low nano-molar concentration range are cytotoxic to several different human cancer cell lines [1,2,3,4,5,6,7]. Further studies revealed that these compounds act as mitocans [8]. Furthermore, conjugates of triterpenes with only one acetyl group in ring A (e.g., derived from oleanolic acid, ursolic acid, glycyrrhetinic acid, betulinic acid, or platanic acid, etc.) are highly cytotoxic but are surpassed in efficacy by those compounds with two or three acetyl groups, such as in maslinic acid [9], madecassic acid [10], tormentic acid [11], euscaphic acid [3], and corosolic acid [12], but also conjugates of asiatic acid [13]. Most recently, an asiatic acid rhodamine hybrid was established as an excellent cytotoxic agent holding cytotoxic activity in a sub-nanomolar concentration [13]. For these particular conjugates, the degree of cytotoxicity was contingent upon the nature of the spacer linked to the carboxyl group located on ring E. These conjugates, formed by combining asiatic acid with rhodamine B, exhibited notable cytotoxicity against human tumor cell lines while demonstrating a high degree of selectivity. Notably, a conjugate holding a homopiperazinyl spacer attached to a tri-acetylated asiatic acid skeleton along with rhodamine B displayed a remarkably low EC_50_ value of 0.8 nM against A2780 ovarian cancer cells. Additional experimentation involving staining revealed that this rhodamine B conjugate acted as a mitocan, inciting apoptosis [13].

Further assessments utilizing 3D spheroid models for colon and breast cancer indicated that these conjugates exhibited activity within the low nanomolar range, displaying the capacity to surmount resistance observed with standard chemotherapeutic agents commonly employed in clinical settings. Consequently, this hybrid engendered cytotoxic responses that were comparable to those elicited by chemotherapy in both sensitive and resistant tumor models. Investigations into mitochondrial function and ATP production stemming from glycolysis and respiration substantiated the characterization of these hybrids as mitocans. Furthermore, these analyses unveiled a rapid disruption of cellular energy metabolism as the principal mechanism of action, setting it apart from conventional chemotherapeutic drugs. This distinct mechanism accounts for such hybrids’ efficacy in overcoming resistance to chemotherapeutic agents [13].

In addition, a first trend was observed showing that those amide-spacered triterpenoid-rhodamine hybrids with two acetyl groups in ring A were more cytotoxic than those with only one acetyl group, and compounds with a 2α, 3β configuration of these acetyl groups were superior to those compounds holding acetyl groups at positions C-2 and C-3 in a different configuration [3]. Thus, the outcomes unveiled a heightened cytotoxicity exhibited by the asiatic acid conjugates in comparison with the analogous counterparts derived from oleanolic, ursolic, or maslinic acid. Remarkably, these effects surpassed the potency of previously documented derivatives originating from betulinic and glycyrrhetinic acid. These findings provide conclusive validation for our initial conjecture, signifying that not only the triterpene framework and the nature of the amide play a role, but the count of acetoxy groups and the specific linkage position of the rhodamine moiety profoundly influence both the cytotoxic potency and the preferential targeting of tumor cells.

It is well known that lipophilic cations exhibit diverse chemical architectures and encompass distinct mechanisms underlying mitochondrial toxicity. For instance, dequalinium chloride functions through the inhibition of NADH-ubiquinone reductase within the mitochondrial respiratory chain, thereby fostering excessive reactive oxygen species (ROS) generation and triggering the opening of the mitochondrial permeability transition (MPT) pore. As mentioned above, the influence of rhodamine-123 manifests in the disruption of mitochondria’s bioenergetic functions, achieved via the inhibition of ATP synthase. The compound denoted as F16 elicits apoptosis by diminishing mitochondrial resistance to the initiation of the calcium-dependent MPT pore. This difference might also apply to substituted rhodamines. Hence, a clear indication as to whether rhodamine B conjugates are superior to those with a rhodamine 101 moiety has not yet been obtained [2]. More recently, it was discovered for piperazinyl-spacered rhodamine conjugates that the substitution pattern on the rhodamine skeleton exerts some effects onto the cytotoxicity of the compounds as well [3]. We have recently shown that the presence of a 1,5-diazacyclooctane spacer (which has a higher degree of molecular flexibility than a piperazinyl or homopiperazinyl residue) leads to hybrids with nanomolar cytotoxicity [4]. However, a systematic study further investigating the dependence of the observed cytotoxicity on the type of cyclic spacer in combination with spacers of different ring sizes is also missing. It has only been shown that the conjugates must contain lipophilic cations [14,15,16,17,18,19,20,21]. While lipophilic cations derived from structurally simple ammonium salts showed only moderate cytotoxicity [22,23], diminished cytotoxicity was also observed for BODIPY conjugates [24], and also for hybrids holding a malachite green [25] scaffold. Conjugates holding an ethylenediamine spacer and a rhodamine B moiety were also inactive [6]. The latter compounds do not form cationic structures under physiological conditions but rather exist as neutral compounds [26]. However, whether the triterpene had an ursane or oleanane backbone does not seem to affect cytotoxicity, as shown by a comparison of analogous of maslinic acid and corosolic acid [3]. Previous research has failed to identify which spacer (piperazine, homopiperazine, or 1,5-diazacyclooctane) is best suited to active compounds with high cytotoxicity.

## 2. Results

**Scheme 1:**
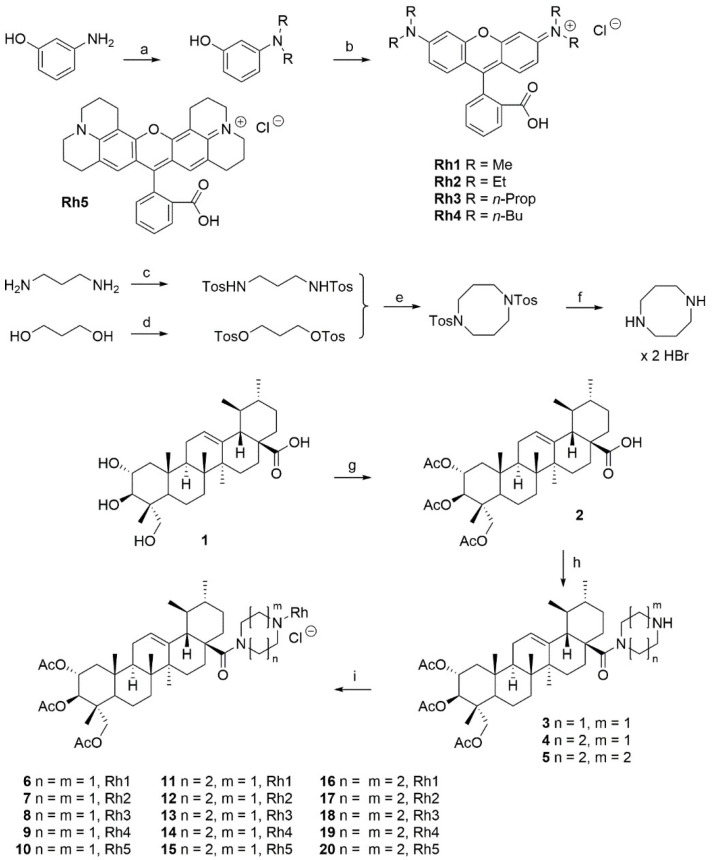
Synthesis of starting material (rhodamines **Rh1**–**Rh4**; structure of **Rh5**) and compounds **2**–**20;** reactions and conditions: (a) DMF, K_2_CO_3_; for R = Me (from MeI, 52%), for R = Et (from EtBr, 67%), for R = *n*-Prop (from *n*-Prop-Br, 61%), for R = *n*-Bu (from n-Bu-Br, 68%), 3–8 h, 21 °C; (b) phthalic anhydride, AlCl_3_ (cat.), 5–60 min, 200 °C: for R = Me 35%, R = Et 45%, R = *n*-Prop 42%, R = *n*-Bu 47%; (c) TosCl, H_2_N-(CH_2_)_3_-NH_2_, no solvent, 80 °C, 30 min, 83%; (d) TosCl, HO-(CH_2_)_3_-OH, pyridine, 0 °C, 30 min, 87%; (e) NaOMe, MeOH, DMF, 80 °C, 12 h, 84%; (f) HBr (33% in glacial acetic acid), 80 °C, 3 h, 92%; (g) Ac_2_O, DCM, NEt_3_, DMAP (cat.), 21 °C, 24 h, 97%; (h) DCM, (COCl)_2_, DMF (cat), NEt_3_; then piperazine (→ **3**, 86%), homopiperazine (→ **4**, 79%), or 1,5-diazocinyl dihydrobromide (→ **5**, 68%); (i) **Rh1**–**Rh5**, (COCl)_2_, NEt_3_, DCM, DMF (cat.), 21 °C, 3 h, then **3**–**5**, NEt_3_, DMAP (cat.), 1 h: **6** (59%), **7** (80%), **8** (57%), **9** (49%), **10** (55%), **11** (49%), **12** (57%), **13** (64%), **14** (58%), **15** (63%), **16** (55%), **17** (60%), **18** (68%), **19** (70%), **20** (64%).

From these premises, it was concluded that asiatic acid (**1**, Scheme 1) should be an ideal starting material for systematic studies. Asiatic acid holds three hydroxyl groups at positions C-2, C-3, and C-23; it is commercially available at reasonable prices. In addition, the two hydroxyl groups in ring A are configured 2α,3β.

Since the amide-bound spacer must be a secondary amine group, piperazine, homopiperazine (perhydro-1,4-diazepane), and the ring-extended analog octahydro-1,5-diazocine (a “bis-homopiperazine”) were selected. While the first two amines are commercially available, the latter is not commercially available but can be synthesized via various routes reported in the literature [27,28,29,30,31,32,33,34,35,36]. Many different routes were tried, but in the end, only one proved to be truly feasible [37]. The tosylation of 1,3-propane-diamine (Scheme 1) and 1,3-propanediol with *p*-toluenesulfonyl chloride gave the corresponding di-tosylates. Their reaction with sodium methoxide in refluxing methanol afforded 1,5-bis(*p*-toluenesulfonyl)-1,5-diazacyclooctane in an 84% isolated yield. Its treatment with HBr (33% in glacial acetic acid) in the presence of an excess of thioanisole [37] afforded octahydro-1,5-diazocine as dihydromide in a 92% isolated yield. This meant that the third spacer to be used in this study was also now readily available and could be prepared in larger quantities.

A further problem arose in the selection of the differently substituted rhodamines. Whilst rhodamine B and rhodamine 101 are commercially available, this is not the case for the analogs that differ in the substituents on the two nitrogen substituents of the rhodamine. The syntheses of the alkyl-substituted rhodamines were carried out as previously reported [13]. Briefly, the reaction of 3-aminophenol with an excess of the corresponding alkyl halide and potassium carbonate in DMF at 100 °C for 3–8 h afforded 3-(dialkylamino)-phenols in a 50–70% yield. Their reaction with phthalic anhydride in the presence of aluminium chloride (catalytic amounts) at 200 °C followed by chromatography (silica gel, CHCl_3_/MeOH mixtures) afforded the rhodamines **Rh1**–**Rh4** violet solids. Although the overall yields obtained for the rhodamines were low to moderate (but in excellent agreement with reported values), no attempts have been made to improve the yields. Significant product loss was observed during the purification of these compounds.

Thus, all the building blocks were now available. Asiatic acid (**1**) was acetylated (Scheme 1) as previously described, and tri-*O*-acetyl-asiatic acid (**2**) was obtained in a 97% yield [13]. The activation of **2** with oxalyl chloride afforded an intermediate acid chloride which was reacted with piperazine, homopiperazine, and its ring-expanded homolog, octahydro-1,5-diazocine, to give amides **3**–**5**. The reaction with the rhodamines **Rh1**–**Rh5** (previously activated with oxalyl chloride) gave the piperazine-derived conjugates **6**–**10**, the homopiperazine derivatives **11**–**15**, and the ring-expanded compounds **16**–**20**. All conjugates exhibited their typical purple color, clearly demonstrating that they are present in a cationic form [6].

**Figure 1:**
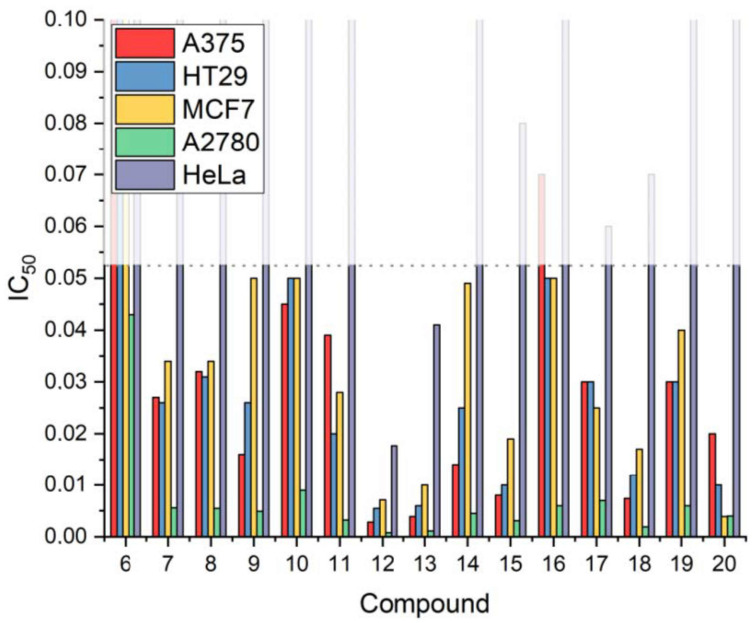
Cytotoxicity of compounds **6**–**20** for different human tumor cell lines.

Sulforhodamine (SRB) assays were performed to evaluate the cytotoxicity of the compounds. First, the cytotoxicity of the different rhodamines was determined; the results of these assays are compiled in Table 1 and Table 2 and depicted in Figure 1.

Thereby methyl- and ethyl (=rhodamine B)-substituted rhodamines **Rh1** and **Rh2** were found to not be cytotoxic within the limits of the assay (EC_50_ > 30 μM) for all human tumor cell lines but also for non-malignant fibroblasts (NIH 3T3). Increased cytotoxicity, however, was observed for the rhodamines carrying propyl or butyl moieties. Thereby, the butyl-substituted rhodamine **Rh4** was more cytotoxic than the propyl-substituted analog **Rh3**. **Rh5** (=rhodamine 101) was about as cytotoxic as the propyl-substituted rhodamine **Rh3**.

**Figure 2:**
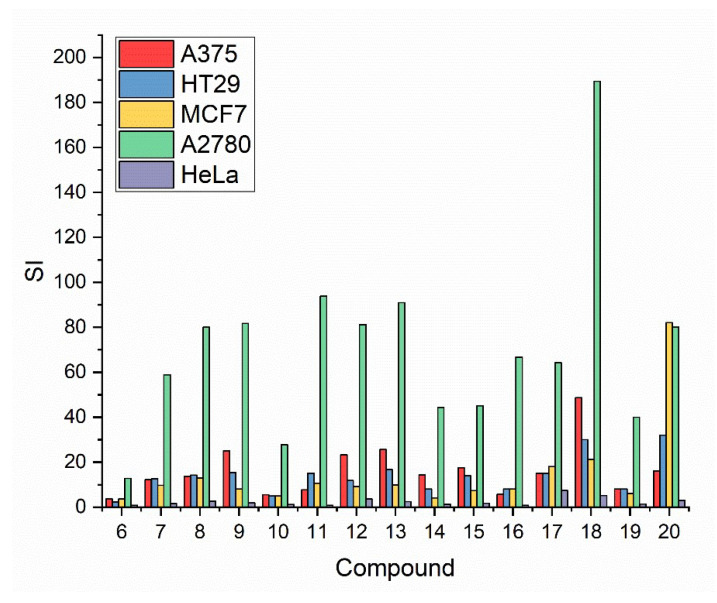
Selectivity index (ratio EC_50,malignantcellline_/EC_50non-malignantNIH3T3_) for compounds **6**–**20**.

As a result, the A2780 cell line (ovarian cancer) proved to be the most sensitive for the piperazinyl spacer compounds **6–10**, with the lowest EC_50_ values determined ranging from 0.043 μM to 0.0049 μM. When compared with non-malignant NIH 3T3 fibroblasts, a selectivity factor (calculated as the ratio EC_50, tumor cell/_EC_50, NIH 3T3_) of 82 was observed, indicating that this compound is more cytotoxic to cancer cells than to non-malignant cells. In this series of compounds, the butyl-substituted rhodamine-conjugated asiatic acid hybrid **9** also proved to be the best for A375 (melanoma) and HT29 (colon adenocarcinoma) cells. A summary of SI values is depicted in Figure 2.

Interestingly, for the homopiperazinyl conjugates **11**–**15,** the compounds that performed best were those holding a rhodamine B (Rh2) unit, and for A2780 cells, a low EC_50_ value of 0.8 nm was determined. The selectivity factor (relative to NIH 3T3) was about 81. A375, HT29, MCF7 (breast adenocarcinoma), and HeLa (cervix carcinoma) cells also responded well to this compound with low EC_50_ values between 0.0028 and 0.0177 μM.

Increasing the ring size of the spacer using the 1,5-diazocinyl spacer changed the dependence of cytotoxicity on the residue attached to the rhodamine core since the rhodamine B derivatives no longer performed best compared with the *N*, *N*-dipropyl substituted compound **18**; therefore, for A2780 cells, an EC_50_ = 1.9 nM was determined, and an excellent selectivity factor of almost 190 was thereby observed.

Rhodamine 101 (**Rh5**) conjugates behaved—more or less—like the alkyl-substituted rhodamine conjugates, and the lowest EC_50_ values were again measured for A2780 cells, and thereby the lowest EC_50_ value was 0.004 μM (selectivity factor = 80).

**Figure 3:**
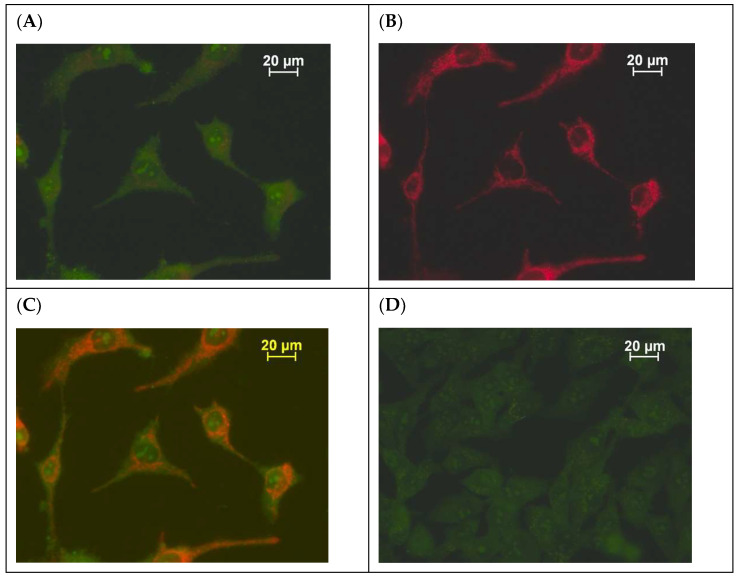
Incubation of A375 cells (48 h) with Mito Tracker green and compound 18: (**A**): detection of Mito Tracker green; (**B**): detection of compound **18**; (**C**): merged pictures (**A**,**B**); (**D**) control.

In conclusion, all human tumor cell lines were shown to be very sensitive to the spacered asiatic acid-rhodamine hybrids, and a significant influence of the substitution pattern of the rhodamine onto the cytotoxic effect was observed. A similar behavior was observed for the influence of the amide spacer linking the triterpenoid skeleton and the rhodamine part. For example, for A2780 cells and propyl substituents, the EC_50_ decreased for the piperazinyl-spacered **8** (5.5 nM) to EC_50_ = 1.1 nM (for **13**) but increased again slightly for **18** (EC_50_ = 1.9 nM). Although it is not possible to generalize which spacer/rhodamine combination performed best across all cell lines, it appears that homopiperazinyl and 1,5-diazocinyl derivatives are superior to those carrying a “simple” piperazinyl moiety. Our previous studies have shown that triterpenoic acid–rhodamine conjugates act as mitocans, shutting down mitochondrial ATP production [13]. This is in excellent agreement with the results of Modico-Napolitano et al. for rhodamine 123 [5]. These researchers showed that the mitochondrial ATPase is the primary biochemical target for rhodamine 123, but they also found that differences in both mitochondrial membrane potential and ATPase sensitivity contribute to selective cytotoxicity for certain cell types in vitro [4,5]. This also applies, for example, to compound **18** (Figure 3). The simultaneous incubation of A375 cells with **18** and MitoTracker green shows that **18** is also preferentially incorporated into the mitochondria.

Investigations employing 3D spheroid models as well as multi-resistant tumor cell lines (such as MDA-MB 231) will be carried out in due course to investigate the potential of these conjugates in more detail.

## 3. Conclusions

A series of piperazinyl-, homopiperazinyl-, and 1,5-diazocinyl-spacered rhodamine conjugates were prepared from asiatic acid, a naturally occurring pentacyclic triterpene. The hybrids were tested for cytotoxic activity in SRB assays with respect to different ring sizes of the spacer and to a different substitution pattern of the rhodamine scaffold. Therefore, compound **12** holding an EC_50_ of 0.8 nM was the most cytotoxic compound of this series but **18** with EC_50_ = 1.9 nM was the most selective compound exhibiting a selectivity factor of almost 190.

## 4. Experimental Section

Experimental equipment: as previously described [2,4] biological assays were performed as previously reported employing cell lines obtained from the Department of Oncology [Martin-Luther-University Halle Wittenberg; they were bought from ATCC [2,4,7]]. Rhodamine 101 B, as well as asiatic acid, were obtained from local vendors and used as received.

For the SRB assay and staining experiments: as previously described [2,4].

### 4.1. Synthesis of 1,5-Diazocinyl Dihydrobromide

This compound was prepared as described in the literature [4,37].

### 4.2. General Procedure for the Synthesis of Triterpenoic Amides ***3***–***5*** (GP A)

Compound **1** (1 eq.) was dissolved in dry DCM (10 mL), and oxalyl chloride (4 eq.), DMF (0.24 eq.) and NEt_3_ (0.24 eq.) were added. The mixture was stirred for 2 h at ambient temperature, the volatiles were removed in vacuo, and the residue was dissolved in dry DCM (10 mL). A solution of the piperazine, homopiperazine or 1,5-diazocinyl dihydromide (3 eq.) in dry DCM, NEt_3_ (1 eq.; 4 eq. when using 1,5-diazocinyl dihydromide) and DMAP (cat.) was added, and stirring was continued until completion of the reaction (as indicated by TLC). The solvent was removed under reduced pressure, and the crude product was subjected to column chromatography to yield compounds **3**–**5**.

### 4.3. General Procedure for Synthesis of Rhodamine B Conjugates ***6***–***20*** (GP B)

Rhodamine **Rh1**–**Rh5** (1.5 eq.), oxalyl chloride (6 eq.), DMF (0.2 eq.), and NEt_3_ (0.2 eq.) in dry DCM (30 mL) were allowed to react as described above (GP A), the volatiles were removed, and the residue was dissolved in dry DCM (30 mL). To this solution, compounds **3**–**5** (1 eq.), NEt_3_ (1.5 eq.), and DMAP (cat.) were added. After stirring for 1 h, the solvent was removed under reduced pressure, and the resulting solid was subjected to column chromatography to yield compounds **6**–**20**, each as a purple solid.

### 4.4. Tri-O-Acetyl-Asiatic Acid [(2α,3β,4α) 2,3,23-Tris(acetyloxy)-urs-12-en-28-oic Acid] *(**2**)*

Acetylation of asiatic acid (**1**, 11.2 g, 21.8 mmol) in dry DCM (250 mL) with Ac_2_O (20.0 mL, 210 mmol) in the presence of NEt_3_ (26 mL, 180 mmol) and catal. DMAP for 24 h followed by usual aq. workup and chromatography (SiO_2_, hexanes/ethyl acetate, 7:3) gave **2** (97%) as a colorless solid; m.p. 160–162 °C (lit.: [13] 160–163 °C); ${\left[\alpha \right]}_{D}^{20}$ = +35.8° (*c* 0.25, CHCl_3_) [lit.: [13] ${\left[\alpha \right]}_{D}^{20}$ = +34.71° (*c* 0.35, CHCl_3_)]; R_F_ = 0.29 (hexanes/ethyl acetate, 7:3); MS (ESI, MeOH): *m*/*z* = 615.2 (15% [M+H]^+^, 637.3 (100%, [M+Na]^+^).

### 4.5. (2α,3β,4α)28-Oxo-28-Piperazin-1-yl-urs-12-ene-2,3,23-Triyl Triacetate *(**3**)*

Following GP A, from **2** (200 mg, 0.32 mmol) and piperazine (85 mg, 0.97 mmol), **3** (190 mg, 86%) was obtained as a colorless solid; m.p. 158–160 °C (lit.: [13] 157–160 °C); ${\left[\alpha \right]}_{D}^{20}$ = +17.20° (*c* 0.31, CHCl_3_), [lit. [13] ${\left[\alpha \right]}_{D}^{20}$ = +17.48° (*c* 0.27, CHCl_3_)]; R_F_ = 0.33 (SiO_2_, CHCl_3_/MeOH, 9:1); MS (ESI, MeOH): *m*/*z* = 683.4 (100%, [M+H]^+^).

### 4.6. (2α,3β,4α)28-(1,4-Diazepan-1-yl)-28-oxo-urs-12-ene-2,3,23-Triyl Triacetate *(**4**)*

Following GP A, from **2** (500 mg, 0.81 mmol) and homopiperazine (210 mg, 2.43 mmol), **4** (445 mg, 79%) was obtained as a colorless solid; m.p. 185–187 °C (lit.: [13] 185.3–186.3 °C)); ${\left[\alpha \right]}_{D}^{20}$ = +14.5° (*c* 0.21, CHCl_3_), [lit.: [13] ${\left[\alpha \right]}_{D}^{20}$ = +14.38° (*c* 0.145, CHCl_3_)]; R_F_ = 0.40 (SiO_2_, CHCl_3_/MeOH, 9:1); MS (ESI, MeOH): *m*/*z* = 697.4 (100%, [M+H]^+^).

### 4.7. (2α,3β,4α)28-(1,5-Diazocin-1-yl)-28-oxo-urs-12-ene-2,3,23-Triyl Triacetate *(**5**)*

Following GP A, from **2** (400 mg, 0.80 mmol) and 1,5-diazocinyl dihydromide (660 mg, 2.4 mmol), **5** (390 mg, 68%) was obtained as a colorless solid; m.p. 187–190 °C (lit. [4] 188–191 °C); R_F_ = 0.36 (CHCl_3_/MeOH, 9:1); ${\left[\alpha \right]}_{D}^{20}$ = −29.7° (*c* 0.02, CHCl_3_) [lit.: [4] ${\left[\alpha \right]}_{D}^{20}$ = -30.2° (*c* 0.015, CHCl_3_)]; MS (ESI, MeOH/CHCl_3_, 4:1): *m*/*z* = 711.2 (100%, [M+H^+^); analysis calcd for C_42_H_66_N_2_O_7_ (711.00): C 70.95, H 9.36, N 3.94; found: C 70.71, H 9.63, N 3.75.

### 4.8. (2α,3β,4α)-9-[2-{[4-(2,3,23-Tris(acetyloxy)-urs-12-en-28-oyl)-Piperazinyl]Carbonyl}Phenyl]-3,6-bis(Dimethylamino)-Xanthylium Chloride *(**6**)*

Following GB B, from **3** (150 mg, 0.22 mmol) followed by chromatography (SiO_2_, CHCl_3_/MeOH, 9:1), **6** (140 mg, 59%) was obtained as a violet solid; m.p. = 248–250 °C; R_f_ = 0.62 (SiO_2_, CHCl_3_/MeOH, 8:2); UV-Vis (MeOH): λ_max_ (log ε) = 552 nm (4.71); IR (ATR): ν = 2923*m*, 2869*w*, 1737*m*, 1630*m*, 1592*vs*, 1534*w*, 1493*m*, 1457*w*, 1406*m*, 1365*m*, 1340*s*, 1231*s*, 1184*vs*, 1124*m*, 1042*m*, 1004*m*, 926*m*, 823*m*, 699*m*, 596*w*, 580*w*, 517*w* cm^−1^; ^1^H NMR (500 MHz, CDCl_3_): δ = 7.71–7.60 (*m*, 2H, 44-H, 46-H), 7.55–7.48 (*m*, 1H, 47-H), 7.39–7.29 (*m*, 1H, 45-H), 7.28–7.22 (*m*, 2H, 51-H), 7.07–6.93 (*m*, 2H, 50-H), 6.86–6.77 (*m*, 2H, 53-H), 5.19–5.08 (*m*, 2H, 12-H, 2-H), 5.04 (*d*, *J* = 10.3 Hz, 1H, 3-H), 3.83 (*d*, *J* = 11.6 Hz, 1H, 24-H_a_), 3.54 (*d*, *J* = 11.9 Hz, 1H, 24-H_b_), 3.43–3.19 (*m*, 8H, 37-H, 38-H, 39-H, 40-H), 3.32 (*s*, 12H, 55-H, 56-H), 2.41–2.22 (*m*, 1H, 18-H), 2.05 (*s*, 3H, 32-H), 2.04–2.00 (*m*, 1H, 1-H_a_), 1.99 (*s*, 3H, 34-H), 1.94 (*s*, 3H, 36-H), 1.93–1.85 (*m*, 3H, 11-H, 16-H_a_), 1.71–1.63 (*m*, 2H, 16-H_b_, 22-H_a_), 1.60–1.54 (*m*, 1H, 9-H), 1.53–1.19 (*m*, 9H, 22-H_b_, 21-H_a_, 7-H_a_, 6-H, 19-H, 5-H, 21-H_b_, 7-H_b_), 1.10 (*d*, *J* = 12.2 Hz, 1H, 1-H_b_), 1.04 (*s*, 4H, 25-H, 15-H_a_), 1.02 (*s*, 4H, 27-H, 15-H_b_), 0.89 (*s*, 4H, 20-H, 30-H), 0.85 (*s*, 3H, 23-H), 0.82–0.79 (*m*, 3H, 29-H), 0.63 (*s*, 3H, 26-H) ppm; ^13^C NMR (126 MHz, CDCl_3_): δ = 175.9 (C-28), 170.9 (C-35), 170.5 (C-31), 170.4 (C-33), 167.7 (C-41), 157.6 (C-54), 156.4 (C-48, C-52), 144.1 (C-13), 135.2 (C-43), 131.8 (C-51), 130.5 (C-42), 130.4 (C-46), 130.4 (C-44), 130.2 (C-45), 127.8 (C-47), 124.7 (C-12), 114.5 (C-50), 114.0 (C-49), 96.9 (C-53), 74.9 (C-3), 70.0 (C-2), 65.3 (C-24), 55.1 (C-18), 48.7 (C-17), 47.7 (C-9), 47.6 (C-5), 43.8 (C-1), 42.2 (C-37, C-38, C-39, C-40), 42.2 (C-14), 42.0 (C-4), 41.2 (C-55, C-56), 39.5 (C-19), 38.7 (C-20), 37.9 (C-8, C-10), 34.2 (C-22), 32.4 (C-7), 30.4 (C-21), 28.1 (C-15), 23.4 (C-11, C-16), 23.4 (C-27), 21.2 (C-36), 21.1 (C-30), 21.0 (C-34), 20.8 (C-32), 17.9 (C-6), 17.4 (C-29), 17.1 (C-25), 16.9 (C-26), 14.0 (C-23) ppm; MS (ESI, MeOH/CHCl_3_): *m*/z = 1052 (100%, [M-Cl]^+^); analysis calcd for C_64_H_834_N_4_O_9_Cl (1087.84): C 70.66, H 7.69, N 5.15; found: C 70.39, H 7.91, N 4.96.

### 4.9. (2α,3β,4α)-9-[2-{[4-(2,3,23-Tris(Acetyloxy)-urs-12-en-28-oyl)-Piperazinyl]Carbonyl}Phenyl]-3,6-Bis(Diethylamino)-Xanthylium Chloride) *(**7**)*

Following GP B, from **3** (70 mg, 0.10 mmol) and rhodamine B (72 mg, 0.15 mmol), **7** (93 mg, 80%) was obtained as a purple solid; m.p. 241–243 °C (lit.: [4] 244–246 °C); R_f_ = 0.28 (SiO_2_, CHCl_3_/MeOH, 9:1); MS (ESI, MeOH): *m*/*z* = 1107.7 (100%, [M-Cl]^+^).

### 4.10. (2α,3β,4α)-9-[2-{[4-(2,3,23-Tris(Acetyloxy)-urs-12-en-28-oyl)-Piperazinyl]Carbonyl}Phenyl]-3,6-Bis(Dipropylamino)-Xanthylium Chloride *(**8**)*

Following GP B, from **3** (150 mg, 0.22 mmol) followed by chromatography (SiO_2_, CHCl_3_/MeOH 9:1) gave **8** (150 mg, 57%) as a violet solid; m.p. 226–228 °C; R_f_ = 0.55 (SiO_2_, CHCl_3_/MeOH 8:2); UV-Vis (MeOH): λ_max_ (log ε) = 563 nm (4.72); IR (ATR): ν = 2926*m*, 2872*w*, 1739*m*, 1633*m*, 1589*vs*, 1545*w*, 1456*m*, 1410*m*, 1366*m*, 1336*s*, 1301*w*, 1230*vs*, 1178*s*, 1132*m*, 1100*m*, 1041*m*, 1004*m*, 962*w*, 940*w*, 918*m*, 825*w*, 758*w*, 597*w*, 576*w*, 506*w* cm^−1^; ^1^H NMR (500 MHz, CDCl_3_): δ = 7.71–7.62 (*m*, 2H, 45-H, 46-H), 7.56–7.48 (*m*, 1H, 47-H), 7.37–7.32 (*m*, 1H, 44-H), 7.31–7.22 (*m*, 2H, 51-H), 7.01–6.94 (*m*, 2H, 50-H), 6.76–6.69 (*m*, 2H, 53-H), 5.19–5.09 (*m*, 2H, 12-H, 2-H), 5.06 (*d*, *J* = 10.3 Hz, 1H, 3-H), 3.83 (*d*, *J* = 11.8 Hz, 1H, 24-H_a_), 3.56 (*d*, *J* = 11.9 Hz, 1H, 24-H_b_), 3.49 (*t*, *J* = 7.9, 7.2 Hz, 8H, 55-H, 56-H), 3.46–3.24 (*m*, 8H, 37-H, 38-H, 39-H, 40-H), 2.39–2.30 (*m*, 1H, 18-H), 2.07 (s, 3H, 32-H), 2.05–2.01 (*m*, 1H, 1-H_a_), 2.00 (*s*, 3H, 34-H), 1.96 (*s*, 3H, 36-H), 1.94–1.87 (*m*, 3H, 11-H, 16-H_a_), 1.76–1.66 (*m*, 10H, 57-H, 59-H, 16-H_b_, 22-H_a_), 1.62–1.56 (*m*, 2H, 9-H, 22-H_b_), 1.50–1.17 (*m*, 8H, 21-H_a_, 7-H_a_, 6-H, 19-H, 5-H, 21-H_b_, 7-H_b_), 1.12–1.08 (*m*, 2H, 1-H_b_, 15-H_a_), 1.05 (*s*, 3H, 25-H), 1.03 (*s*, 4H, 27-H, 15-H_b_), 1.00 (*t*, *J* = 7.4 Hz, 12H, 58-H), 0.92–0.90 (*m*, 4H, 20-H, 30-H), 0.86 (*s*, 3H, 23-H), 0.84–0.81 (*m*, 3H, 29-H), 0.67 (*s*, 3H, 26-H) ppm; ^13^C NMR (126 MHz, CDCl_3_): δ = 176.1 (C-28), 171.0 (C-35), 170.6 (C-31), 170.4 (C-33), 167.9 (C-41), 157.8 (C-54), 156.3 (C-48), 156.2 (C-52), 143.2 (C-13), 135.1 (C-43), 132.3 (C-51), 130.8 (C-42), 130.5 (C-44), 130.4 (C-45), 130.4 (C-46), 127.8 (C-47), 124.7 (C-12), 114.6 (C-50), 114.0 (C-49), 96.6 (C-53), 74.9 (C-3), 70.0 (C-2), 65.4 (C-24), 54.7 (C-18), 53.9 (C-55, C-56), 49.0 (C-17), 47.7 (C-9), 47.6 (C-5), 47.4 (C-37, C-38, C-39, C-40), 43.8 (C-1), 42.2 (C-14), 42.0 (C-4), 39.5, 39.3 (C-19), 38.7 (C-20), 37.9 (C-8, C-10), 34.3 (C-22), 32.6 (C-7), 30.5 (C-21), 28.1 (C-15), 23.4 (C-11, C-16), 23.4 (C-27), 21.3 (C-36), 21.2 (C-30), 21.0 (C-34), 20.9 (C-32), 20.7 (C-57, C-59), 18.0 (C-6), 17.4 (C-29), 17.1 (C-25), 17.0 (C-26), 14.0 (C-23), 11.4 (C-58, C-60) ppm; MS (ESI, MeOH): *m*/z = 1164.5 (100%, [M-Cl]^+^); analysis calcd for C_72_H_99_N_4_O_9_Cl (1200.03): C 72.06, H 8.32, N 4.67; found: C 71.85, H 8.53, N 4.40.

### 4.11. (2α,3β,4α)-9-[2-{[4-(2,3,23-Tris(Acetyloxy)-urs-12-en-28-oyl)-Piperazinyl]Carbonyl}Phenyl]-3,6-Bis(Dibutylamino)-Xanthylium Chloride *(**9**)*

Following GP B, from **3** (200 mg, 0.29 mmol) followed by chromatography (SiO_2_, CHCl_3_/MeOH, 9:1), **9** (180 mg, 0.14 mmol, 49%) was obtained as a violet solid; m.p. 222–226 °C; R_f_ = 0.44 (SiO_2_, CHCl_3_/MeOH, 8:2); UV-Vis (MeOH): λ_max_ (log ε) = 567 nm (4.91); IR (ATR): ν = 2929*m*, 2870*w*, 1740*m*, 1633*w*, 1587*vs*, 1528*w*, 1506*w*, 1462*m*, 1429*w*, 1411*s*, 1394*m*, 1366*m*, 1340*s*, 1290*m*, 1219*vs*, 1188*w*, 1176*s*, 1133*m*, 1109*m*, 1042*m*, 1004*m*, 962*w*, 922*m*, 823*w*, 755*w*, 732*w*, 705*w*, 664*w*, 641*w*, 597*w*, 509*w* cm^−1^; ^1^H NMR (500 MHz, CDCl_3_): δ = 7.69–7.64 (*m*, 2H, 45-H, 46-H), 7.55–7.50 (*m*, 1H, 47-H), 7.35–7.30 (*m*, 1H, 44-H), 7.28–7.21 (*m*, 2H, 51-H), 7.07–6.91 (*m*, 2H, 50-H), 6.70 (*s*, 2H, 53-H), 5.15 (*s*, 1H, 12-H), 5.13–5.09 (*m*, 1H, 2-H), 5.04 (*d*, *J* = 10.3 Hz, 1H, 3-H), 3.81 (*d*, *J* = 11.7 Hz, 1H, 24-H_a_), 3.60–3.55 (*m*, 1H, 24-H_b_), 3.54–3.48 (*m*, 8H, 55-H, 59-H), 3.39–3.24 (*m*, 8H, 37-H, 38-H, 39-H, 40-H), 2.37–2.29 (*m*, 1H, 18-H), 2.05 (*s*, 3H, 32-H), 2.04–2.01 (*m*, 1H, 1-H_a_), 1.99 (*s*, 3H, 34-H), 1.94 (*s*, 3H, 36-H), 1.92–1.86 (*m*, 4H, 11-H, 16-H), 1.75–1.70 (*m*, 1H, 22-H_a_), 1.69–1.62 (*m*, 8H, 56-H, 60-H), 1.60–1.55 (*m*, 1H, 9-H), 1.50–1.19 (*m*, 17H, 22-H_b_, 21-H_a_, 57-H, 61-H, 7-H_a_, 6-H, 19-H, 5-H, 21-H_b_, 7-H_b_), 1.10 (*d*, *J* = 11.9 Hz, 1H, 1-H_b_), 1.04 (*s*, 3H, 25-H), 1.02 (*s*, 5H, 27-H, 15-H), 0.96 (*t*, *J* = 6.8 Hz, 12H, 58-H, 62-H), 0.93–0.92 (*m*, 1H, 20-H), 0.90 (*s*, 3H, 30-H), 0.85 (*s*, 3H, 23-H), 0.81 (*d*, *J* = 6.3 Hz, 3H, 29-H), 0.66 (*s*, 3H, 26-H) ppm; ^13^C NMR (126 MHz, CDCl_3_): δ = 175.9 (C-28), 170.9 (C-35), 170.5 (C-31), 170.4 (C-33), 167.8 (C-41), 157.7 (C-54), 156.1 (C-48), 156.0 (C-52), 143.9 (C-13), 135.0 (C-43), 132.3 (C-51), 130.7 (C-42), 130.5 (C-44), 130.4 (C-46), 130.4 (C-45), 127.8 (C-47), 124.7 (C-12), 114.5 (C-50), 113.9 (C-49), 96.5 (C-53), 74.9 (C-3), 70.0 (C-2), 65.3 (C-24), 55.2 (C-18), 52.0 (C-55, C-59), 47.7 (C-9), 47.6 (C-5), 47.4 (C-37, C-38), 43.8 (C-1), 42.3 (C-39, C-40), 42.2 (C-14), 42.0 (C-4, C-17), 39.5 (C-19), 38.7 (C-20), 37.9 (C-8, C-10), 34.2 (C-22), 32.6 (C-7), 30.5 (C-21), 29.6 (C-56, C-60), 28.1 (C-15), 23.4 (C-11, C-16), 23.4 (C-27), 21.2 (C-30), 21.1 (C-36), 20.9 (C-34), 20.8 (C-32), 20.3 (C-57, C-61), 18.0 (C-6), 17.4 (C-29), 17.1 (C-25), 16.9 (C-26), 14.0 (C-23), 13.9 (C-58, C-62) ppm; MS (ESI, MeOH): *m*/z = 1219.4 (100%, [M-Cl]^+^); analysis calcd for C_76_H_107_N_4_O_9_Cl (1256.16): C 72.67, H 8.59, N 4.46; found: C 72.49, H 8.75, N 4.20.

### 4.12. (2α,3β,4α)-2,3,23-Triacetoxy-28-[3-(2,3,6,7,12,13,16,17-Octahydro-1H,5H,11H,15H-Pyrido[3.2.1-Ij]Pyrido[1″,2″,3″:1′,8′]Quinolino[6′,5′:5,6]Pyrano[2,3-F]Quinoline-4-Ium-9-Yl)Benzoyl]-Piperazin-1-Yl]-28-Oxo-Olean-12-Ene Chloride *(**10**)*

Following GP B, from **3** (150 mg, 0.22 mmol) followed by chromatography (SiO_2_, CHCl_3_/MeOH, 9:1), **10** (144 mg, 55%) was obtained as a violet solid; m.p. 244–247 °C; R_f_ = 0.38 (SiO_2_, CHCl_3_/MeOH, 8:2); UV-Vis (MeOH): λ_max_ (log ε) = 581 nm (4.69); IR (ATR): ν = 2924*m*, 2867*w*, 1739*s*, 1630*m*, 1595*s*, 1546*w*, 1493*m*, 1458*w*, 1446*w*, 1364*s*, 1296*s*, 1233*vs*, 1196*s*, 1182*s*, 1097*s*, 1035*s*, 1004*m*, 962*w*, 773*w*, 735*w*, 641*w*, 622*w*, 598*w*, 422*w* cm^−1^; ^1^H NMR (500 MHz, CDCl_3_): δ = 7.70–7.62 (*m*, 2H, 45-H, 46-H), 7.55–7.46 (*m*, 1H, 47-H), 7.30–7.27 (*m*, 1H, 44-H), 6.72–6.62 (*m*, 2H, 50-H), 5.19–5.15 (*m*, 1H, 12-H), 5.12 (*td*, *J* = 11.1, 4.5 Hz, 1H, 2-H), 5.05 (*d*, *J* = 10.3 Hz, 1H, 3-H), 3.83 (*d*, *J* = 11.7 Hz, 1H, 24-H_a_), 3.69–3.21 (*m*, 17H, 24-H_b_, 57-H, 58-H, 37-H, 38-H, 39-H, 40-H), 3.08–2.92 (*m*, 4H, 55-H), 2.78–2.60 (*m*, 4H, 60-H), 2.45–2.25 (*m*, 1H, 18-H), 2.14–2.08 (*m*, 4H, 56-H), 2.06 (*s*, 3H, 32-H), 2.04–2.01 (*m*, 1H, 1-H_a_), 1.99 (*s*, 3H, 34-H), 1.95 (*s*, 3H, 36-H), 1.93–1.86 (*m*, 7H, 59-H, 11-H, 16-H_a_), 1.74–1.64 (*m*, 2H, 22-H_a_, 16-H_b_), 1.62–1.50 (*m*, 2H, 9-H, 22-H_b_), 1.46–1.14 (*m*, 8H, 21-H_a_, 7-H_a_, 6-H, 19-H, 5-H, 21-H_b_, 7-H_b_), 1.14–1.07 (*m*, 2H, 1-H_b_, 15-H_a_), 1.06 (*s*, 3H, 25-H), 1.03 (*s*, 4H, 27-H, 15-H_b_), 0.96–0.92 (*m*, 1H, 20-H), 0.92–0.89 (*m*, 3H, 30-H), 0.86 (*s*, 3H, 29-H), 0.84–0.80 (*m*, 3H, 23-H), 0.67 (*s*, 3H, 26-H) ppm; ^13^C NMR (126 MHz, CDCl_3_): δ = 175.9 (C-28), 170.9 (C-35), 170.5 (C-31), 170.4 (C-33), 168.0 (C-41), 153.1 (C-54,C- 62), 152.1 (C-48), 151.3 (C-52, C-72), 143.9 (C-13), 134.9 (C-43), 131.8 (C-42), 130.8 (C-44), 130.3 (C-46), 129.9 (C-45), 127.6 (C-47), 126.7 (C-50, C-70), 124.8 (C-12), 123.7 (C-51, C-71), 113.3 (C-49, C-61), 105.5 (C-53, C-73), 74.9 (C-3), 70.0 (C-2), 65.3 (C-24), 55.2 (C-18), 51.1 (C-58, C-83), 50.6 (C-57, C-85), 49.0 (C-17), 47.7 (C-9), 47.6 (C-5), 43.8 (C-1), 42.2 (C-14), 42.2 (C-37, C-38, C-39, C-40), 42.0 (C-4), 39.5 (C-19), 38.7 (C-20), 37.9 (C-8, C-10), 34.3 (C-22), 32.6 (C-7), 30.5 (C-21), 28.1 (C-15), 27.7 (C-60, C-81), 23.5 (C-27), 23.4 (C-11, C-16), 21.3 (C-30), 21.1 (C-36), 21.0 (C-34), 20.8 (C-32), 20.7 (C-59, C-82), 20.0 (C-55, C-87), 19.8 (C-56, C-86), 18.0 (C-6), 17.4 (C-29), 17.1 (C-25), 16.9 (C-26), 14.0 (C-23) ppm; MS (ESI, MeOH/CHCl_3_): *m*/z = 1156.3 (100%, [M-Cl]^+^); analysis calcd for C_68_H_78_N_4_O_5_Cl (1040.47): C 78.50, H 8.43, N 5.36; found: C 78.34, H 8.59, N 5.24.

### 4.13. (2α,3β,4α)-9-[2-{[4-(2α,3β,23-Tris(Acetyloxy)-urs-12-en-28-oyl)-Homopiperazinyl]Carbonyl}Phenyl]-3,6-Bis(Dimethylamino)-Xanthylium Chloride *(**11**)*

Following GP B, from **4** (200 mg, 0.29 mmol) followed by chromatography (SiO_2_, CHCl_3_/MeOH, 9:1), **11** (156 mg, 49%) was obtained as a violet solid; m.p. 258–260 °C R_f_ = 0.38 (SiO_2_, CHCl_3_/MeOH, 8:2); UV-Vis (MeOH): λ_max_ (log ε) = 553 nm (4.70); IR (ATR): ν = 2925*m*, 2870*w*, 1738*m*, 1592*vs*, 1534*w*, 1493*m*, 1406*m*, 1365*m*, 1341*s*, 1304*w*, 1231*s*, 1185*s*, 1135*m*, 1042*m*, 1032*m*, 963*w*, 926*m*, 823*w*, 700*m*, 602*w*, 580*w*, 516*w* cm^−1^; ^1^H NMR (400 MHz, CDCl_3_): δ = 7.69–7.57 (*m*, 2H, 45-H, 47-H), 7.47–7.38 (*m*, 1H, 48-H), 7.34–7.28 (*m*, 1H, 46-H), 7.26–7.11 (*m*, 2H, 52-H), 7.02–6.72 (*m*, 4H, 54-H, 51-H), 5.23–5.09 (*m*, 2H, 12-H, 2-H), 5.05 (*d*, *J* = 10.3 Hz, 1H, 3-H), 3.82 (*d*, *J* = 11.7 Hz, 1H, 24-H_a_), 3.55 (*d*, *J* = 11.9 Hz, 1H, 24-H_b_), 3.51–2.79 (*m*, 22H, 56-H, 57-H, 37-H, 38-H, 39-H, 40-H, 41-H), 2.49–2.32 (*m*, 1H, 18-H), 2.05 (*s*, 3H, 32-H), 2.04–2.01 (*m*, 1H, 1-H_a_), 1.99 (*s*, 3H, 34-H), 1.95 (*s*, 3H, 36-H), 1.94–1.82 (*m*, 3H, 11-H, 16-H_a_), 1.82–1.62 (*m*, 2H, 16-H_a_, 22-H_a_), 1.63–1.54 (*m*, 1H, 9-H), 1.50–1.17 (*m*, 9H, 22-H_b_, 21-H_a_, 7-H_a_, 6-H, 19-H, 5-H, 21-H_b_, 7-H_b_), 1.11 (*d*, *J* = 12.0 Hz, 1H, 1-H_b_), 1.05 (*s*, 3H, 25-H), 1.02 (*s*, 4H, 27-H, 15-H_a_), 1.00–0.96 (*m*, 2H, 20-H, 15-H_a_), 0.95–0.88 (*m*, 3H, 30-H), 0.85 (*s*, 6H, 23-H, 29-H), 0.69 (*s*, 3H, 26-H) ppm; ^13^C NMR (126 MHz, CDCl_3_): δ = 173.3 (C-28), 171.0 (C-35), 170.6 (C-31), 170.5 (C-33), 168.1 (C-42), 157.7 (C-55), 157.6 (C-49), 157.5 (C-53), 144.9 (C-13), 135.0 (C-44), 132.0 (C-52), 130.5 (C-43), 130.3 (C-47), 130.2 (C-45), 129.7 (C-46), 126.9 (C-48), 124.8 (C-12), 114.1 (C-51), 113.9 (C-50), 96.8 (C-54), 75.0 (C-3), 70.0 (C-2), 65.4 (C-24), 55.4 (C-18), 49.0 (C-17), 47.8 (C-9), 47.7 (C-5), 43.8 (C-1), 42.4 (C-14), 42.0 (C-4), 41.4 (C-37, C-38, C-39, C-40, C-41), 41.2 (C-56, C-57), 39.4 (C-19), 38.8 (C-20), 37.9 (C-8, C-10), 34.0 (C-22), 32.4 (C-7), 30.6 (C-21), 27.9 (C-15), 23.4 (C-27), 23.4 (C-11, C-16), 21.3 (C-30), 21.2 (C-36), 21.0 (C-34), 20.9 (C-32), 18.0 (C-6), 17.5 (C-29), 17.1 (C-25), 17.0 (C-26), 14.0 (C-23) ppm; MS (ESI, MeOH/CHCl_3_): *m*/z = 1065.5 (100%, [M-Cl]^+^); analysis calcd for C_65_H_85_N_4_O_9_Cl (1101.86): C 70.85, H 7.78, N 5.08; found: C 70.59, H 7.93, N 4.88.

### 4.14. (2α,3β,4α)-9-[2-{[4-(2α,3β,23-Tris(Acetyloxy)-urs-12-en-28-oyl)-Homopiperazinyl]Carbonyl}Phenyl]-3,6-Bis(Diethylamino)-Xanthylium Chloride *(**12**)*

Following GP B, from **4** (180 mg, 0.26 mmol) followed by chromatography (SiO_2_, CHCl_3_/MeOH, 9:1), **12** (224 mg, 64%) was obtained as a violet solid; m.p. 253–257 °C (lit.:[4] 254–258 °C; R_f_ = 0.30 (SiO_2_, CHCl_3_/MeOH, 8:1); MS (ESI, MeOH/CHCl_3_): *m*/*z* = 1121.4 (100%, [M-Cl]^+^).

### 4.15. (2α,3β,4α)-9-[2-{[4-(2α,3β,23-Tris(Acetyloxy)-urs-12-en-28-oyl)-Homopiperazinyl]Carbonyl}Phenyl]-3,6-Bis(Dipropylamino)-Xanthylium Chloride *(**13**)*

Following GP B, from **4** (200 mg, 0.29 mmol) followed by chromatography (SiO_2_, CHCl_3_/MeOH, 9:1), **13** (224 mg, 64%) was obtained as a violet solid; m.p. 224–225 °C; R_f_ = 0.33 (SiO_2_, CHCl_3_/MeOH, 8:2); UV-Vis (MeOH): λ_max_ (log ε) = 566 nm (4.91); IR (ATR): ν = 2928*m*, 2873*w*, 1739*m*, 1627*m*, 1587*vs*, 1528*w*, 1507*w*, 1470*m*, 1412*s*, 1365*m*, 1337*s*, 1301*m*, 1230*vs*, 1177*s*, 1133*m*, 1100*m*, 1041*m*, 1032*m*, 997*w*, 963*w*, 939*w*, 919*w*, 824*w*, 780*w*, 757*w*, 597*w*, 575*w*, 507*w* cm^−1^; ^1^H NMR (500 MHz, CDCl_3_): δ = 7.64–7.57 (*m*, 2H, 46-H, 47-H), 7.46–7.37 (*m*, 1H, 48-H), 7.30–7.15 (*m*, 3H, 45-H, 52-H), 6.78–6.67 (*m*, 2H, 54-H), 5.20–5.15 (*m*, 1H, 2-H), 5.12 (*td*, *J* = 11.0, 4.7 Hz, 2H, 12-H), 5.04 (*d*, *J* = 10.3 Hz, 1H, 3-H), 4.16–3.86 (*m*, 4H, 37-H, 38-H), 3.80 (*d*, *J* = 11.7 Hz, 1H, 24-H_a_), 3.54 (*d*, *J* = 11.8 Hz, 1H, 24-H_b_), 3.53–3.38 (*m*, 8H, 56-H, 59-H), 3.34–2.97 (*m*, 8H, 39-H, 40-H), 2.49–2.35 (*m*, 1H, 18-H), 2.05 (*s*, 3H, 32-H), 2.03–2.00 (*m*, 1H, 1-H_a_), 1.98 (*s*, 3H, 34-H), 1.94 (*s*, 3H, 36-H), 1.93–1.83 (*m*, 3H, 11-H, 16-H_a_), 1.79–1.65 (*m*, 10H, 57-H, 60-H, 16-H_b_, 22-H_a_), 1.62–1.53 (*m*, 1H, 22-H_b_, 9-H), 1.42–1.18 (*m*, 10H, 21-H_a_, 7-H_a_, 6-H, 19-H, 5-H, 21-H_b_, 7-H_b_, 41-H), 1.10–1.07 (*m*, 1H, 1-H_b_), 1.04 (s, 3H, 25-H), 1.01 (*s*, 4H, 27-H, 15-H_a_), 1.00–0.96 (*m*, 13H, 58-H, 61-H, 20-H), 0.93–0.88 (*m*, 4H, 30-H, 15-H_b_), 0.85 (*s*, 6H, 23-H, 29-H), 0.68 (*s*, 3H, 26-H) ppm; ^13^C NMR (126 MHz, CDCl_3_): δ = 176.6 (C-28), 170.9 (C-35), 170.5 (C-33), 170.4 (C-31), 168.1 (C-42), 157.8 (C-55), 156.3 (C-49), 156.2 (C-53), 142.6 (C-13), 136.0 (C-44), 132.6 (C-52), 130.7 (C-43), 130.2 (C-47), 130.2 (C-45), 129.7 (C-46), 126.9 (C-48), 124.6 (C-12), 114.9 (C-51), 113.8 (C-50), 96.6 (C-54), 77.4, 77.2, 76.9, 75.0 (C-3), 70.0 (C-2), 65.4 (C-24), 55.8 (C-18), 53.9 (C-56, C-59), 53.8 (C-37, C-38, C-39, C-40, C-41), 48.9 (C-17), 47.8 (C-9), 47.7 (C-5), 43.8 (C-1), 42.4 (C-14), 42.0 (C-4), 39.4 (C-19), 38.8 (C-20), 37.9 (C-8, C-10), 34.0 (C-22), 32.7 (C-7), 30.5 (C-21), 28.0 (C-15), 23.4 (C-11, C-16), 23.4 (C-27), 21.3 (C-30), 21.1 (C-36), 21.0 (C-34), 20.8 (C-32), 20.8 (C-57, C-60), 18.0 (C-6), 17.5 (C-29), 17.1 (C-25), 17.0 (C-26), 14.0 (C-23), 11.4 (C-58, C-61) ppm; MS (ESI, MeOH/CHCl_3_): *m*/z = 1178.4 (100%, [M-Cl]^+^); analysis calcd for C_73_H_101_N_4_O_9_Cl (1214.08): C 72.22, H 8.39, N 4.61; found: C 71.97, H 8.48, N 4.39.

### 4.16. (2α,3β,4α)-9-[2-{[4-(2α,3β,23-Tris(Acetyloxy)-urs-12-en-28-oyl)-Homopiperazinyl]Carbonyl}Phenyl]-3,6-Bis(Dibutylamino)-Xanthylium Chloride *(**14**)*

Following GP B, from **4** (284 mg, 0.4 mmol) followed by chromatography (SiO_2_, CHCl_3_/MeOH, 9:1), **14** (2198 mg, 58%) was obtained as a violet solid; m.p. 210–213 °C; R_f_ = 0.36 (SiO_2_, CHCl_3_/MeOH, 8:2); UV-Vis (MeOH): λ_max_ (log ε) = 567 nm (4.94); IR (ATR): ν = 2927*m*, 2870*w*, 1741*m*, 1626*w*, 1588*vs*, 1528*w*, 1507*w*, 1462*m*, 1412*s*, 1394*w*, 1366*m*, 1339*s*, 1291*m*, 1220*s*, 1187*s*, 1177*s*, 1133*m*, 1109*m*, 1043*m*, 1033*m,* 963*w*, 921*m*, 823*w*, 756*w*, 704*w*, 597*w* cm^−1^; ^1^H NMR (500 MHz, CDCl_3_): δ = 7.68–7.54 (*m*, 2H, 46-H, 47-H), 7.46–7.36 (*m*, 1H, 48-H), 7.29–7.25 (*m*, 2H, 45-H), 7.25–7.14 (*m*, 2H, 52-H), 7.09–6.98 (*m*, 2H, 51-H), 6.79–6.64 (*m*, 2H, 54-H), 5.18–5.15 (*m*, 1H, 12-H), 5.12 (*td*, *J* = 11.0, 4.7 Hz, 1H, 2-H), 5.03 (*d*, *J* = 10.3 Hz, 1H, 3-H), 4.26–2.89 (*m*, 20H, 24-H, 37-H, 38-H, 39-H, 40-H, 41-H, 56-H, 60-H), 2.49–2.33 (*m*, 1H, 18-H), 2.04 (*s*, 3H, 32-H), 2.03–2.00 (*m*, 1H, 1-H_a_), 1.98 (*s*, 3H, 34-H), 1.94 (*s*, 3H, 36-H), 1.92–1.82 (*m*, 4H, 11-H, 16-H), 1.81–1.72 (*m*, 2H, 22-H), 1.71–1.61 (*m*, 8H, 57-H, 61-H), 1.61–1.54 (*m*, 1H, 9-H), 1.48–1.18 (*m*, 16H, 58-H, 62-H, 21-H_a_, 7-H_a_, 6-H, 19-H, 5-H, 21-H_b_, 7-H_b_), 1.12–1.07 (*m*, 1H, 1-H_b_), 1.04 (*m*, 5H, 25-H, 15-H), 1.01 (*s*, 3H, 27-H), 0.96 (*t*, *J* = 7.2 Hz, 13H, 59-H, 63-H, 20-H), 0.93–0.88 (*m*, 3H, 30-H), 0.84 (*s*, 6H, 23-H, 29-H), 0.68 (*s*, 3H, 26-H) ppm; ^13^C NMR (126 MHz, CDCl_3_): δ = 176.4 (C-28), 170.9 (C-35), 170.5 (C-31) 170.4 (C-33), 168.0 (C-42), 157.8 (C-55), 156.1 (C-49), 156.0 (C-53), 143.9 (C-13), 136.0 (C-44), 132.6 (C-52), 130.2 (C-43), 130.2 (C-45), 129.8 (C-47), 129.7 (C-46), 126.9 (C-48), 124.6 (C-12), 114.7 (C-51), 113.8 (C-50), 96.4 (C-54), 75.0 (C-3), 70.0 (C-2), 65.4 (C-24), 55.5 (C-18), 52.1 (C-37, C-38, C-39, C-40, C-41), 52.0 (C-56, C-60), 49.0 (C-17), 47.8 (C-9), 47.7 (C-5), 43.8 (C-1), 42.4 (C-14), 42.0 (C-4), 39.4 (C-19), 38.8 (C-20), 37.9 (C-8, C-10), 34.0 (C-22), 32.6 (C-7), 30.5 (C-21), 29.6 (C-57, C-62), 27.6 (C-15), 23.4 (C-11, C-16), 23.4 (C-27), 21.3 (C-30), 21.1 (C-36), 20.9 (C-34), 20.8 (C-32), 20.3 (C-58, C-63), 17.9 (C-6), 17.5 (C-29), 17.1 (C-25), 17.0 (C-26), 14.0 (C-23), 13.9 (C-59, C-64) ppm; MS (ESI, MeOH/CHCl_3_): *m*/z = 1234.3 (100%, [M-Cl]^+^); analysis calcd for C_77_H_109_N_4_O_9_Cl (1270.19): C 72.81, H 8.65, N 4.41; found: C 72.61, H 8.86, N 4.21.

### 4.17. (2α,3β,4α)-2,3,23-Triacetoxy-28-[3-(2,3,6,7,12,13,16,17-Octahydro-1H,5H,11H,15H-Pyrido[3.2.1-Ij]Pyrido[1″,2″,3″:1′,8′]Quinolino[6′,5′:5,6]Pyrano[2,3-F]Quinoline-4-Ium-9-Yl)Benzoyl]-1,4-Diazepan-1-Yl]-28-Oxo-Olean-12-Ene Chloride *(**15**)*

Following GP B, from **4** (115 mg, 0.17 mmol) followed by chromatography (SiO_2_, CHCl_3_/MeOH, 9:1), **15** (104 mg, 0.1 mmol, 63%) was obtained as a violet solid; m.p. 287–289 °C; R_f_ = 0.19 (SiO_2_, CHCl_3_/MeOH, 9:1); UV-Vis (MeOH): λ_max_ (log ε) = 582 nm (4.82); IR (ATR): ν = 2924*m*, 2866*w*, 1738*s*, 1621*m*, 1595*vs*, 1545*w*, 1493*m*, 1458*w*, 1445*w*, 1435*w*, 1363*s*, 1294*vs*, 1232*s*, 1179*vs*, 1100*s*, 1034*s*, 962*w*, 743*m*, 640*w*, 623*w*, 598*w*, 420*m* cm^−1^; ^1^H NMR (500 MHz, CDCl_3_): δ = 7.72–7.52 (*m*, 2H, 46-H, 47-H), 7.47–7.34 (*m*, 1H, 48-H), 7.25–7.20 (*m*, 1H, 45-H), 6.79–6.55 (*m*, 2H, 51-H), 5.22–5.17 (*m*, 1H, 12-H), 5.14 (*td*, *J* = 11.1, 4.5 Hz, 1H, 2-H), 5.05 (*d*, *J* = 10.3 Hz, 1H, 3-H), 4.50–3.89 (*m*, 4H, 37-H, 38-H), 3.82 (*d*, *J* = 11.6 Hz, 1H, 24-H_a_), 3.69–3.15 (*m*, 13H, 24-H_b_, 39-H, 40-H, 58-H, 59-H), 3.05–2.95 (*m*, 4H, 56-H), 2.80–2.63 (*m*, 4H, 61-H), 2.49–2.39 (*m*, 1H, 18-H), 2.15–2.08 (*m*, 4H, 57-H), 2.06 (*s*, 3H, 32-H), 2.04–2.02 (*m*, 1H, 1-H_a_), 2.00 (*s*, 3H, 34-H), 1.96 (*s*, 3H, 36-H), 1.94–1.84 (*m*, 5H, 60-H, 11-H_a_), 1.85–1.64 (*m*, 4H, 11-H_b_, 16-H, 22-H_a_), 1.63–1.56 (*m*, 2H, 9-H, 22-H_b_), 1.51–1.14 (*m*, 10H, 21-H_a_, 7-H_a_, 6-H, 19-H, 5-H, 21-H_b_, 7-H_b_, 41-H), 1.14–1.08 (*m*, 1H, 1-H_b_), 1.06 (*s*, 4H, 25-H, 15-H_a_), 1.02 (*s*, 4H, 27-H, 15-H_b_), 0.97–0.92 (*m*, 1H, 20-H), 0.92–0.88 (*m*, 3H, 30-H), 0.86 (*s*, 6H, 23-H, 29-H), 0.70 (*s*, 3H, 26-H) ppm; ^13^C NMR (126 MHz, CDCl_3_): δ = 175.5 (C-28), 170.8 (C-35), 170.4 (C-31), 170.3 (C-33), 168.0 (C-42), 152.9 (C-55), 151.9 (C-49), 151.3 (C-53), 143.8 (C-13), 135.0 (C-44), 131.8 (C-43), 130.4 (C-45), 129.6 (C-46), 129.5 (C-47), 126.7 (C-48), 126.6 (C-51), 124.7 (C-12), 123.7 (C-52), 113.4 (C-50), 105.1 (C-54), 74.9 (C-3), 69.9 (C-2), 65.3 (C-24), 55.6 (C-18), 51.0 (C-37, C-38, C-39, C-40), 51.0 (C-59), 50.6 (C-58), 48.9 (C-17), 47.7 (C-9), 47.6 (C-5), 43.7 (C-1), 42.0 (C-4), 41.9 (C-14), 39.4 (C-19), 38.7 (C-20), 37.8 (C-8, C-10), 34.3 (C-22), 32.5 (C-7), 30.5 (C-21), 29.7 (C-41), 27.6 (C-15), 27.6 (C-61), 23.4 (C-27), 23.3 (C-11, C-16), 21.2 (C-30), 21.0 (C-36), 20.9 (C-34), 20.7 (C-32), 20.6 (C-60), 19.9 (C-56), 19.7 (C-57), 17.8 (C-6), 17.4 (C-29), 17.3 (C-25), 17.0 (C-26), 13.9 (C-23) ppm; MS (ESI, MeOH/CHCl_3_): *m*/z = 1169.7 (100%, [M-Cl]^+^); analysis calcd for C_69_H_89_N_4_O_5_Cl (1054.49): C 78.59, H 8.51, N 5.31; found: C 78.22, H 8.79, N 5.06.

### 4.18. (2α,3β,4α)-9-[2-[{4-(2α,3β,23-Tris(Acetyloxy)-urs-12-en-28-oyl)-1,5-Diazocan-1-Yl]Carbonyl}Phenyl]-3,6-Bis(Dimethylamino)-Xanthylium Chloride *(**16**)*

Following GP B, from **5** (670 mg, 0.94 mmol) followed by chromatography (SiO_2_, CHCl_3_/MeOH, 9:1), **16** (523 mg, 55%) was obtained as a violet solid; m.p. 224–226 °C; R_f_ = 0.50 (SiO_2_, CHCl_3_/MeOH, 8:2); UV-Vis (MeOH): λ_max_ (log ε) = 556 nm (4.72); IR (ATR): ν = 2922*s*, 2852*m*, 1741*m*, 1624*w*, 1593*vs*, 1534*w*, 1508*w*, 1493*m*, 1437*w*, 1407*m*, 1365*m*, 1343*s*, 1285*w*, 1231*s*, 1185*vs*, 1135*m*, 1085*w*, 1043*m*, 1033*m*, 925*m*, 820*m*, 757*w*, 699*m*, 518*w*, 492*w* cm^−1^; ^1^H NMR (500 MHz, CDCl_3_): δ = 7.70–7.58 (*m*, 2H, 46-H, 48-H), 7.57–7.46 (*m*, 1H, 49-H), 7.37–7.27 (*m*, 3H, 47-H, 53-H), 7.06–6.87 (*m*, 2H, 52-H), 6.84–6.65 (*m*, 2H, 55-H), 5.23–5.17 (*m*, 1H, 12-H), 5.14 (*td*, *J* = 11.2, 5.3 Hz, 1H, 2-H), 5.06 (*d*, *J* = 10.3 Hz, 1H, 3-H), 3.82 (*d*, *J* = 11.8 Hz, 1H, 24-H_a_), 3.56 (*d*, *J* = 11.9 Hz, 1H, 24-H_b_), 3.52–2.83 (*m*, 8H, 37-H, 38-H, 41-H, 42-H), 3.40–3.29 (*m*, 12H, 57-H, 58-H), 2.48–2.38 (*m*, 1H, 18-H), 2.07 (*s*, 3H, 32-H), 2.05–2.02 (*m*, 1H, 1-H_a_), 2.00 (*s*, 3H, 34-H), 1.96 (*s*, 3H, 36-H), 1.94–1.73 (*m*, 4H, 11-H, 16-H), 1.63–1.55 (*m*, 1H, 9-H), 1.54–1.14 (*m*, 15H, 21-H_a_, 7-H_a_, 22-H_a_, 6-H, 39-H, 40-H, 19-H, 5-H, 22-H_b_, 21-H_b_, 7-H_b_, 15-H_a_), 1.10 (*s*, 2H, 1-H_b_, 15-H_b_), 1.08–1.02 (*m*, 6H, 25-H, 27-H), 1.01–0.95 (*m*, 1H, 20-H), 0.93–0.89 (*m*, 3H, 30-H), 0.86 (*s*, 3H, 23-H), 0.85–0.82 (*m*, 3H, 29-H), 0.72 (*s*, 3H, 26-H) ppm; ^13^C NMR (126 MHz, CDCl_3_): δ = 175.8 (C-28), 171.0 (C-35), 170.6 (C-31), 170.5 (C-33), 167.9 (C-43), 157.7 (C-56), 157.5 (C-54), 156.4 (C-50), 145.0 (C-13), 136.8 (C-45), 131.2 (C-53), 130.6 (C-44), 130.4 (C-46, C-48), 130.1 (C-47), 127.9 (C-49), 124.6 (C-12), 114.4 (C-52), 114.1 (C-51), 96.9 (C-55), 77.4, 77.2, 76.9, 76.9, 75.0 (C-3), 70.1 (C-2), 65.4 (C-24), 55.6 (C-18), 48.0 (C-17), 47.8 (C-9), 47.7 (C-5), 43.9 (C-1), 42.7 (C-37, C-38, C-41, C-42), 42.2 (C-14), 42.0 (C-4), 41.4 (C-57, C-58), 39.4 (C-19), 38.8 (C-20), 38.0 (C-8, C-10), 34.1 (C-22), 32.0 (C-7), 29.8 (C-21), 27.4 (C-15), 25.0, 23.4 (C-11, C-16), 23.4 (C-27), 22.8 (C-39, C-40), 21.2 (C-36), 21.0 (C-34), 20.9 (C-32), 18.0 (C-6), 17.5 (C-29), 17.2 (C-25), 17.0 (C-26), 14.0 (C-23) ppm; MS (ESI, MeOH/CHCl_3_): *m*/z = 1079.5 (100%, [M-Cl]^+^); analysis calcd for C_66_H_87_N_4_O_9_Cl (1115.89): C 71.04, H 7.86, N 5.02; found: C 70.86, H 8.03, N 4.77.

### 4.19. (2α,3β,4α)-9-[2-[{4-(2α,3β,23-Tris(Acetyloxy)-urs-12-en-28-oyl)-1,5-Diazocan-1-Yl]Carbonyl}Phenyl]-3,6-Bis(Diethylamino)-Xanthylium Chloride *(**17**)*

Following GP B, from **5** (300 mg, 0.4 mmol) followed by chromatography (SiO_2_, CHCl_3_/MeOH, 9:1), **17** (188 mg, 60%) was obtained as a purple solid; m.p. 222–227 °C (lit: [4] 223–226 °C); R_f_ = 0.42 (SiO_2_, CHCl_3_/MeOH, 9:1); MS (ESI, MeOH/CHCl_3_): *m*/z = 1036.3 (100%, [M-Cl]^+^); analysis calcd for C_70_H_95_N_4_O_9_Cl (1172.00): C 71.74, H 8.17, N 4.78; found: C 71.48, H 8.22, N 5.37.

### 4.20. (2α,3β,4α)-9-[2-[{4-(2α,3β,23-Tris(Acetyloxy)-urs-12-en-28-oyl)-1,5-Diazocan-1-Yl]Carbonyl}Phenyl]-3,6-Bis(Dipropylamino)-Xanthylium Chloride *(**18**)*

Following GP B, from **5** (500 mg, 0.7 mmol) followed by chromatography (SiO_2_, CHCl_3_/MeOH, 9:1), **18** (612 mg, 68%) was obtained as a violet solid; m.p. 207–209 °C; R_f_ = 0.53 (SiO_2_, CHCl_3_/MeOH, 8:2); UV-Vis (MeOH): λ_max_ (log ε) = 564 nm (4.47); IR (ATR): ν = 2927*m*, 2873*w*, 1740*s*, 1696*w*, 1589*s*, 1528*w*, 1504*w*, 1457*m*, 1432*w*, 1412*m*, 1367*m*, 1338*m*, 1301*w*, 1230*vs*, 1194*w*, 1178*m*, 1133*m*, 1101*w*, 1042*m*, 1032*m*, 964*w*, 939*w*, 918*w*, 825*w*, 641*w*, 598*w*, 508*w* cm^−1^; ^1^H NMR (400 MHz, CDCl_3_): δ = 7.70–7.57 (*m*, 2H, 47-H, 48-H), 7.57–7.47 (*m*, 1H, 49-H), 7.39–7.27 (*m*, 3H, 46-H, 53-H), 7.15–6.88 (*m*, 2H, 52-H), 6.79–6.62 (*m*, 2H, 55-H), 5.27–5.18 (*m*, 1H, 12-H), 5.14 (*td*, *J* = 11.2, 5.5 Hz, 1H, 2-H), 5.06 (*d*, *J* = 10.3 Hz, 1H, 3-H), 3.82 (*d*, *J* = 11.6 Hz, 1H, 24-H_a_), 3.76–2.76 (*m*, 17H, 24-H_b_, 57-H, 37-H, 38-H, 41-H, 42-H), 2.48–2.31 (*m*, 1H, 18-H), 2.07 (s, 3H, 32-H), 2.05–2.02 (*m*, 1H, 1-H_a_), 2.00 (*s*, 3H, 34-H), 1.96 (*s*, 3H, 36-H), 1.95–1.84 (*m*, 4H, 11-H, 16-H), 1.81–1.65 (*m*, 8H, 58-H), 1.65–1.56 (*m*, 1H, 9-H), 1.56–1.22 (*m*, 14H, 22-H, 21-H_a_, 7-H_a_, 39-H, 40-H, 6-H, 19-H, 5-H, 21-H_b_, 7-H_b_), 1.15–1.10 (*m*, 1H, 1-H_b_), 1.10–1.06 (*m*, 4H, 25-H, 15-H_a_), 1.04 (*s*, 4H, 27-H, 15-H_b_), 1.01 (*t*, *J* = 7.0 Hz, 12H, 59-H), 0.97–0.94 (*m*, 1H, 20-H), 0.92 (*s*, 3H, 30-H), 0.88–0.85 (*m*, 3H, 23-H), 0.85 (*s*, 3H, 29-H), 0.77–0.69 (*m*, 3H, 26-H) ppm; ^13^C NMR (101 MHz, CDCl_3_): δ = 176.1 (C-28), 171.0 (C-35), 170.6 (C-33), 170.5 (C-31), 168.2 (C-43), 157.8 (C-56), 156.3 (C-50), 156.2 (C-54), 144.0 (C-13), 136.1 (C-45), 132.4 (C-53), 130.7 (C-44), 130.3 (C-48), 130.0 (C-46), 129.5 (C-47), 127.3 (C-49), 124.7 (C-12), 115.0 (C-52), 114.0 (C-51), 96.4 (C-55), 75.0 (C-3), 70.1 (C-2), 65.4 (C-24), 55.2 (C-18), 53.9 (C-37, C-38, C-41, C-42), 53.9 (C-57#, C-57), 49.2 (C-17), 47.8 (C-5), 47.7 (C-9), 43.9 (C-1), 42.0 (C-14), 42.0 (C-4), 39.5 (C-19), 39.5 (C-8), 38.8 (C-20), 38.0 (C-10), 34.0 (C-22), 32.7 (C-7), 30.7 (C-21), 29.8 (C-39, C-40), 28.3 (C-15), 23.5 (C-16), 23.5 (C-27), 23.4 (C-11), 21.4 (C-30), 21.2 (C-32), 21.0 (C-34), 20.9 (C-36), 20.9 (C-58#, C-58), 18.0 (C-6), 17.5 (C-29), 17.4 (C-25), 17.2 (C-26), 14.0 (C-23), 11.5 (C-59#, 59) ppm; MS (ESI, MeOH/CHCl_3_): *m*/z = 1191.3 (100%, [M-Cl]^+^); analysis calcd for C_74_H_103_N_4_O_9_Cl (1228.11): C 72.37, H 8.45, N 4.56; found: C 72.11, H 8.64, N 4.40.

### 4.21. (2α,3β,4α)-9-[2-[{4-(2α,3β,23-Tris(Acetyloxy)-urs-12-en-28-oyl)-1,5-Diazocan-1-Yl]Carbonyl}Phenyl]-3,6-Bis(Dibutylamino)-Xanthylium Chloride *(**19**)*

Following GP B, from **5** (400 mg, 0.56 mmol) followed by chromatography (SiO_2_, CHCl_3_/MeOH, 9:1), **19** (520 mg, 70%) was obtained as a violet solid;.m.p. 215–218 °C; R_f_ = 0.43 (SiO_2_, CHCl_3_/MeOH, 8:2); UV-Vis (MeOH): λ_max_ (log ε) = 566 nm (4.79); IR (ATR): ν = 2930*m*, 2871*w*, 1741*m*, 1633*w*, 1589*vs*, 1528*w*, 1461*m*, 1412*s*, 1394*w*, 1367*m*, 1338*s*, 1292*m*, 1221*vs*, 1178*s*, 1133*m*, 1109*m*, 1043*m*, 964*w*, 921*m*, 825*w*, 757*w*, 597*w* cm^−1^; ^1^H NMR (500 MHz, CDCl_3_): δ = 7.68–7.54 (*m*, 2H, 47-H, 48-H), 7.52–7.46 (*m*, 1H, 49-H), 7.31–7.18 (*m*, 3H, 53-H, 46-H), 7.04–6.76 (*m*, 2H, 52-H), 6.74–6.57 (*m*, 2H, 55-H), 5.19–5.14 (*m*, 1H, 12-H), 5.11 (*td*, *J* = 10.9, 4.9 Hz, 1H, 2-H), 5.03 (*d*, *J* = 10.3 Hz, 1H, 3-H), 3.79 (*d*, *J* = 11.7 Hz, 1H, 24-H_a_), 3.67–2.76 (*m*, 17H, 24-H_b_, 57-H, 37-H, 38-H, 41-H, 42-H), 2.41–2.33 (*m*, 1H, 18-H), 2.04 (*s*, 3H, 32-H), 2.02–1.99 (*m*, 1H, 1-H_a_), 1.97 (*s*, 3H, 34-H), 1.93 (*s*, 3H, 36-H), 1.91–1.80 (*m*, 3H, 11-H, 16-H_a_), 1.79–1.71 (*m*, 1H, 16-H_b_), 1.69–1.59 (*m*, 8H, 58-H), 1.59–1.54 (*m*, 1H, 9-H), 1.50–1.16 (*m*, 19H, 59-H, 22-H, 7-H_a_, 21-H_a_, 6-H, 19-H, 39-H, 40-H, 5-H, 7-H_b_, 21-H_b_, 15-H_a_), 1.09–1.05 (*m*, 2H, 1-H_b_, 15-H_b_), 1.03 (*s*, 3H, 25-H), 1.01 (*s*, 3H, 27-H), 0.95 (*t*, J = 6.1 Hz, 13H, 60-H, 20-H), 0.90–0.86 (*m*, 3H, 30-H), 0.83 (*s*, 3H, 23-H), 0.82–0.78 (*m*, 3H, 29-H), 0.69 (*s*, 3H, 26-H) ppm; ^13^C NMR (126 MHz, CDCl_3_): δ = 176.1 (C-28), 170.9 (C-35), 170.5 (C-31), 170.4 (C-33), 168.0 (C-43), 157.7 (C-56), 156.1 (C-50), 156.1 (C-54), 144.1 (C-13), 136.6 (C-45), 132.2 (C-53), 130.2 (C-48), 130.1 (C-46), 129.4 (C-44), 129.3 (C-47), 127.4 (C-49), 124.5 (C-12), 113.8 (C-52), 113.5 (C-51), 96.3 (C-55), 74.9 (C-3), 70.0 (C-2), 65.3 (C-24), 55.4 (C-18), 52.0 (C-57, C-57), 52.0 (C-37, C-38, C-41, C-42), 49.0 (C-17), 47.7 (C-9), 47.6 (C-5), 43.8 (C-1), 42.1 (C-14), 41.9 (C-4), 39.8 (C-39, C-40), 39.4 (C-19), 38.7 (C-20), 37.9 (C-10), 37.9 (C-8), 34.3 (C-22), 32.6 (C-7), 30.6 (C-21), 29.7 (C-58#, C-58), 28.1 (C-15), 23.4 (C-11, C-16), 23.3 (C-27), 21.3 (C-30), 21.1 (C-36), 20.9 (C-34), 20.8 (C-32), 20.2 (C-59#, C-59), 17.9 (C-6), 17.4 (C-29), 17.1 (C-25), 17.0 (C-26), 14.0 (C-23), 13.9 (C-60, 61) ppm; MS (ESI, MeOH/CHCl_3_): *m*/z = 1247.3 (100%, [M-Cl]^+^); analysis calcd for C_78_H_111_N_4_O_9_Cl (1284.22): C 72.95, H 8.71, N 4.36; found: C 72.70, H 8.96, N 4.17.

### 4.22. (2α,3β,4α)-2,3,23-Triacetoxy-28-[3-(2,3,6,7,12,13,16,17-Octahydro-1H,5H,11H,15H-Pyrido[3.2.1-Ij]Pyrido[1″,2″,3″:1′,8′]Quinolino[6′,5′:5,6]Pyrano[2,3-F]Quinoline-4-Ium-9-Yl)Benzoyl]-1,5-Diazocan-1-Yl]-28-Oxo-Olean-12-Ene Chloride *(**20**)*

Following GP B, from **5** (200 mg, 0.3 mmol) followed by chromatography (SiO_2_, CHCl_3_/MeOH, 9:1), **20** (232 mg, 64%)was obtained as a violet solid; m.p. 194–196 °C (lit.: [4] 193–196 °C); R_f_ = 0.42 (SiO_2_, CHCl_3_/MeOH, 9:1); MS (ESI, MeOH/CHCl_3_): *m*/z = 1084.6 (100%, [M-Cl]^+^); analysis calcd for C_70_H_91_N_4_O_5_Cl (1068.52): C 78.69, H 8.58, N 5.24; found: C 78.32, H 8.86, N 5.09. 

## Figures and Tables

**Table 1:** Cytotoxicity of rhodamines **Rh1**–**Rh5** (in μM) from SRB assays after 72 h of treatment: averaged from three independent experiments each in triplicate; confidence interval CI = 95%. Human tumor cell lines: A375 (melanoma), HT29 (colorectal carcinoma), MCF7 (breast adenocarcinoma), HeLa (cervical adenocarcinoma), A2780 (ovarian carcinoma), NIH 3T3 (non-malignant fibroblasts, murine); cut-off of the assay 30 μM; n.d. not determined; doxorubicin (**DX**) has been used as a positive standard.

	A375	HT29	MCF7	A2780	HeLa	NIH 3T3
**Rh1**	>30	>30	>30	>30	>30	>30
**Rh2**	>30	>30	>30	>30	>30	>30
**Rh3**	8.2 ± 0.2	9.3 ± 0.4	6.4 ± 0.4	6.7 ± 0.7	8.0 ± 0.7	12.0 ± 0.7
**Rh4**	4.3 ± 0.1	4.9 ± 0.3	3.08 ± 0.06	3.4 ± 0.1	4.3 ± 0.3	4.3 ± 0.1
**Rh5**	11.2 ± 1.8	18.5 ± 1.8	8.2 ± 0.8	8.0 ± 1.5	11.8 ± 1.1	11.9 ± 1.3
**DX**	n.d.	0.9 ± 0.2	1.1 ± 0.3	0.02 ± 0.01	n.d.	11.9 ± 1.3

**Table 2:** Cytotoxicity of conjugates **6**–**20** (in μM) from SRB assays after 72 h of treatment: averaged from three independent experiments each in triplicate; confidence interval CI = 95%. Human tumor cell lines: A375 (melanoma), HT29 (colorectal carcinoma), MCF7 (breast adenocarcinoma), HeLa (cervical adenocarcinoma), A2780 (ovarian carcinoma), NIH 3T3 (non-malignant fibroblasts, murine); cut-off of the assay 30 μM; n.d. not determined; doxorubicin (**DX**) has been used as a positive standard.

	A375	HT29	MCF7	A2780	HeLa	NIH 3T3
**6**	0.15 ± 0.008	0.23 ± 0.08	0.15 ± 0.03	0.043 ± 0.008	0.70 ± 0.2	0.55 ± 0.06
**7**	0.027 ± 0.009	0.026 ± 0.009	0.034 ± 0.009	0.0056 ± 0.0006	0.20 ± 0.1	0.33 ± 0.08
**8**	0.032 ± 0.003	0.031 ± 0.007	0.034 ± 0.005	0.0055 ± 0.0005	0.16 ± 0.05	0.44 ± 0.09
**9**	0.016 ± 0.003	0.026 ± 0.008	0.05 ± 0.01	0.0049 ± 0.0007	0.21 ± 0.05	0.40 ± 0.1
**10**	0.045 ± 0.004	0.05 ± 0.02	0.05 ± 0.01	0.009 ± 0.002	0.22 ± 0.05	0.25 ± 0.05
**11**	0.039 ± 0.003	0.02 ± 0.01	0.028 ± 0.005	0.0032 ± 0.0004	0.40 ± 0.1	0.30 ± 0.1
**12**	0.0028 ± 00005	0.0055 ± 0.0023	0.0071 ± 0.0021	0.0008 ± 0.0001	0.0177 ± 0.0049	0.0650 ± 0.0262
**13**	0.0039 ± 0.0006	0.006 ± 0.001	0.010 ± 0.001	0.0011 ± 0.0001	0.041 ± 0.009	0.10 ± 0.05
**14**	0.014 ± 0.001	0.025 ± 0.005	0.049 ± 0.006	0.0045 ± 0.0004	0.15 ± 0.05	0.20 ± 0.1
**15**	0.008 ± 0.001	0.010 ± 0.006	0.019 ± 0.006	0.0031 ± 0.0005	0.08 ± 0.03	0.14 ± 0.05
**16**	0.07 ± 0.01	0.05 ± 0.02	0.05 ± 0.01	0.006 ± 0.001	0.50 ± 0.2	0.40 ± 0.09
**17**	0.03 ± 0.01	0.03 ± 0.01	0.025 ± 0.02	0.007 ± 0.002	0.06 ± 0.01	0.45 ± 0.02
**18**	0.0074 ± 0.0009	0.012 ± 0.004	0.017 ± 0.002	0.0019 ± 0.0002	0.07 ± 0.02	0.36 ± 0.05
**19**	0.03 ± 0.01	0.03 ± 0.01	0.04 ± 0.005	0.006 ± 0.002	0.17 ± 0.08	0.24 ± 0.08
**20**	0.02 ± 0.01	0.01 ± 0.005	0.0039 ± 0.002	0.004 ± 0.001	0.11 ± 0.06	0.32 ± 0.02
**DX**	n.d.	0.9 ± 0.2	1.1 ± 0.3	0.02 ± 0.01	n.d.	11.9 ±1.3

## Data Availability

The data presented in this study are available on request from the corresponding author.

## Sample Availability

Samples of the compounds are available from the authors.
